# RFX3 loss disrupts non-coding RNA networks in iPSC-derived pancreatic progenitors

**DOI:** 10.1016/j.gendis.2025.101959

**Published:** 2025-11-27

**Authors:** Noura Aldous, Aldana Alnesf, Ahmed K. Elsayed, Bushra Yasin Abohalawa, Nehad M. Alajez, Essam M. Abdelalim

**Affiliations:** a Pluripotent Stem Cell Disease Modelling Lab, Translational Medicine Department, Research Branch, Sidra Medicine, Doha 26999, Qatar; b College of Health and Life Sciences, Hamad Bin Khalifa University (HBKU), Qatar Foundation (QF), Doha 34110, Qatar; c Translational Oncology Research Centre (TORC), Qatar Biomedical Research Institute (QBRI), Hamad Bin Khalifa University (HBKU), Qatar Foundation (QF), Doha 34110, Qatar

Regulatory factor X 3 (RFX3), a member of the highly conserved RFX family of transcription factors, has recently been identified to be essential for human pancreatic endocrine development and β-cell function. Recently, we showed that loss of RFX3 during pancreatic differentiation of human induced pluripotent stem cells (iPSCs) disrupts endocrine gene regulation, reduces islet hormone-secreting cells, impairs β-cell function, and notably leads to increased cell death and aberrant specification toward enterochromaffin cells.^1^ Non-coding RNAs (ncRNAs), including microRNAs (miRNAs) and long ncRNAs (lncRNAs), play critical roles in regulating pancreatic development, especially in the formation and function of pancreatic islets and β-cells.^2^ While RFX3’s role in gene regulation is established, its impact on ncRNA networks during pancreatic differentiation remains poorly understood.

In the current study, we leveraged our previously established *RFX3*-deficient human iPSC model to investigate how RFX3 loss altered the expression profiles of miRNAs and lncRNAs at the pancreatic progenitor (PP) stage^1^ (Fig. S1A–S1C; Table S1, S2). By integrating mRNA, miRNA, and lncRNA expression data, we identified differentially expressed transcripts (DEGs, DEmiRs, and DElncRNAs) and constructed competing endogenous RNA (ceRNA) networks targeting key pancreatic genes at PPs (Fig. S1A). miRNA-sequencing analysis using RNA samples of iPSC-derived PPs revealed 70 significantly up-regulated DEmiRs (log_2_ [fold change] > 0.5, *P*-value < 0.05) and 52 significantly down-regulated DEmiRs (log_2_ [fold change] < −0.5, *P*-value < 0.05) (Fig. 1A; Fig. S1D). In contrast, RNA-sequencing analysis revealed 178 significantly down-regulated (log_2_ [fold change] < –0.5, *P*-value < 0.05) and 64 significantly up-regulated (log_2_ [fold change] > 0.5, *P*-value < 0.05) DElncRNAs in *RFX3* knockout PPs compared with wild-type PPs (Fig. 1B; Fig. S1E). Multiple DEmiRs and DElncRNAs were validated using quantitative real-time PCR (Fig. S2; Table S3). Given our recent findings that showed substantial impairments of islet development in the absence of RFX3, our current study focused on the up-regulated DEmiRs, many of which were predicted to regulate key PP genes and lncRNAs (Fig. 1C).

**Figure 1:**
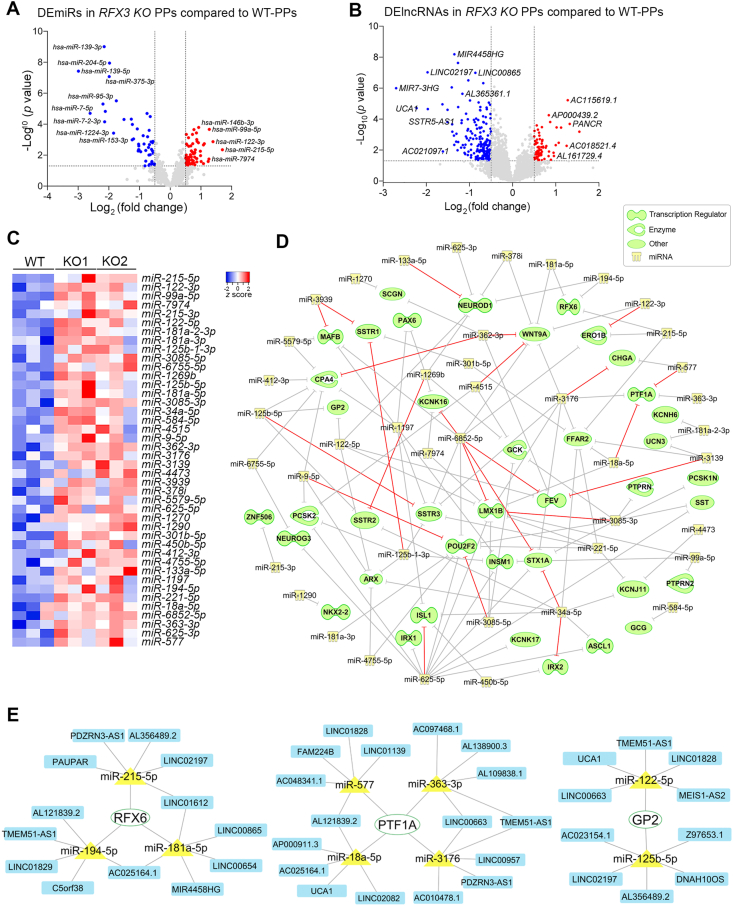
RFX3 loss disrupts non-coding RNA regulatory networks in induced pluripotent stem cell (iPSC)-derived pancreatic progenitors (PPs). **(A, B)** Volcano plots showing significantly down-regulated (blue) and up-regulated (red) differentially expressed miRNAs (A) and lncRNAs (B) in PPs lacking RFX3. **(C)** Heatmap of selected up-regulated DEmiRs predicted to target key pancreatic genes (*n* = 3). **(D)** Target prediction analysis of up-regulated DEmiRs targeting key down-regulated pancreatic differentially expressed genes (DEGs). The red lines indicate a high prediction confidence level, while the grey lines indicate a moderate prediction confidence level. **(E)** Representative miRNA-mRNA-lncRNA correlation networks in RFX3-deficient PPs. The networks illustrate predicted interactions between up-regulated DEmiRs, down-regulated DElncRNAs, and down-regulated DEGs. miRNA target prediction was performed using Ingenuity Pathway Analysis (IPA) and miRanda for DEGs and DElncRNAs, respectively. Selected PP markers were used to construct ceRNA networks. Networks were generated using Cytoscape software.

To further study the impact of DEmiRs in PPs lacking RFX3, we employed the miRNA target filter in Ingenuity Pathway Analysis (IPA) software to integrate the miRNA-sequencing data with our recently published mRNA-sequencing dataset.^1^ This revealed 61 significantly up-regulated DEmiRs (log_2_ [fold change] > 0.5, *P*-value < 0.05) that were predicted to target 333 down-regulated DEGs in *RFX3* knockout PPs. Among these, we selected 40 DEGs, crucial for pancreatic development and/or function, and were significantly impacted by *RFX3* knockout (Fig. 1D). Target prediction analysis revealed that these 40 down-regulated DEGs were targeted by 42 up-regulated DEmiRs (Fig. 1D; Table S4). Of note, *NEUROD1* was a target for multiple up-regulated DEmiRs, including *miR-122-5p*, *miR-1269b, miR-378i*, *miR-133a-5p*, *miR-194-5p*, and *miR-625-3p*. Moreover, *PTF1A* was predicted to be targeted by *miR-3176*, *miR-18a-5p*, *miR-363-3p*, and *miR-577*.

Beyond transcription factors, several up-regulated DEmiRs were predicted to target genes involved in diverse pancreatic islet and β-cell functions. For example, *ERO1B* was a predicted target of *miR-215-5p*, *miR-122-3p*, and *miR-6852-5p*, while *GCK* was targeted by *miR-3085-5p*, *miR-1269b*, *miR-3085-3p*, and *miR-4515*. Also, *UCN3* was predicted to be targeted by *miR-181a-2-3p* and *miR-3139*, and *KCNK1*6 by miR*-7974*, *miR-1269b*, and *miR-6852-5p*. Interestingly, *SST* and its receptors (*SSTRs*), including *SSTR1*, *SSTR2,* and *SSTR3*, were predicted to be targeted by several up-regulated DEmiRs. Specifically, *miR-3085-3p* and *miR-625-5p* were predicted to target *SST*, *miR-125b-1-3p* was predicted to target both *SSTR1* and *SSTR3*, while *miR-125b-5p* was predicted to target both *SSTR2* and *SSTR3.* IPA analysis identified 18 DEmiRs with high-confidence predictions targeting 17 down-regulated DEGs. For instance, *miR-6852-5p* was highly predicted to target *KCNK16*, *LMX1B*, *STX1A*, and *FEV*. Also, *miR-3939* was predicted to target *MAFB* and *SSTR1*, while *miR-362-3p* was predicted to target *WNT9A* and *CPA4*. Importantly, *PTF1A* was a highly predicted target by two DEmiRs, *miR-577* and *miR-18a-5p*. In addition, *miR-3176* was predicted to target *CHGA*, *miR-122-3p* targeted *ERO1B*, and *miR-625-5p* targeted *ISL1*.

To further integrate our findings, we conducted a target prediction analysis to explore the potential interactions and correlations between DEmiRs and DElncRNAs in *RFX3* knockout PPs. We identified 41 up-regulated DEmiRs that were linked to 161 down-regulated DElncRNAs with a confidence interaction score ≥140 and predicted binding energy ≤ –1 kcal/mol. For example, *MIR7-3HG*, which was the most down-regulated DElncRNA in *RFX3* knockout PPs, was correlated to *miR-6755-5p*, *miR-1269b*, *miR-125b-5p*, *miR-3085-3p*, *miR-34a-5p*, *miR-4515*, *miR-584-5p*, *miR-3139*, *miR-378i*, *miR-412-3p*, *miR-4755-5p*, *miR-194-5p*, *miR-221-5p*, and *miR-6852-5p*. Similarly, *UCA1* lncRNA was predicted to interact with *miR-7974*, *miR-215-3p*, *miR-122-5p*, *miR-181a-2-3p*, *miR-3085-5p*, *miR-6755-5p*, *miR-125b-5p*, *miR-3085-3p*, *miR-34a-5p*, *miR-4515*, *miR-584-5p*, *miR-9-5p*, *miR-3139*, *miR-625-5p*, *miR-1270*, *miR-1290*, *miR-4755-5p*, *miR-133a-5p*, *miR-1197*, *miR-194-5p*, *miR-18a-5p*, and *miR-6852-5p*.

Due to a lack of a validated database or datasets for directly predicting DElncRNAs-targeted DEGs, we explored the potential regulation of DEGs mediated by DElncRNAs through DEmiRs. lncRNAs have been identified to display multiple functional capabilities, including their ability to act as molecular decoys for miRNA by competing with mRNA for miRNA binding.^3^ We combined our analysis of DEmiR–DElncRNA interactions and constructed a mRNA-miRNA-lncRNA network by identifying DElncRNAs and DEGs that share binding to the same DEmiRs. As mentioned above, we focused on a set of 40 down-regulated DEGs, which were highly impacted by the RFX3 loss. From these 40 DEGs, we selected a subset of genes with well-established roles in pancreatic development to construct specific ceRNA networks. These key genes included *RFX6*, *PTF1A*, and *GP2*, on which we focused on the top 5 DElncRNAs with the highest stringent alignment and binding energy scores (Fig. 1E). *RFX6*, a critical regulator of pancreatic endocrine specification, was predicted to be targeted by three up-regulated DEmiRs: *miR-181a-5p*, *miR-194-5p*, and *miR-215-5p*. *PTF1A*, a marker for pancreatic exocrine identity, was targeted by four DEmiRs, including *miR-577*, *miR-363-3p*, *miR-18a-5p*, and *miR-3176*. *GP2*, a specific surface marker of PPs, was targeted by *miR-122-5p* and *miR-125b-5p*. Moreover, multiple important pancreatic endocrine genes, including *FEV*, *INSM1*, *IRX1*, *IRX2*, *CHGA*, *NEUROG3*, *NEUROD1*, *NKX2.2*, *PAX6*, and *MAFB*, were also shown to be regulated by multiple DEmiRs and DElncRNAs (Fig. S3).

We examined potential overlaps between ncRNA profiles in tumors involving RFX3 and our RFX3-deficient PP dataset. For instance, *circRFX3*, up-regulated in gliomas, sponges *miR-1179* to increase vasodilator-stimulated phosphoprotein (VASP) expression.^4^ While we also observed *miR-1179* down-regulation, VASP levels remained unchanged, suggesting alternative regulatory mechanisms in our model. Similarly, *miR-577*, which acts as either a tumor suppressor or oncogene depending on the cancer type, was up-regulated in our dataset. However, its reported target, STAT3, showed no change in expression, unlike findings in non-small cell lung cancer, where *RFX3-AS1* sponges *miR-577* to activate STAT3.^5^ These discrepancies underscore the tissue- and context-specific nature of ncRNA regulation, indicating that mechanisms identified in cancer may not directly apply to developmental systems like the pancreas.

In summary, using integrative transcriptomic profiling of mRNAs, miRNAs, and lncRNAs in RFX3-deficient iPSC-derived PPs, we identified widespread dysregulation of both coding and non-coding RNAs. Up-regulated DEmiRs were predicted to target essential pancreatic transcription factors and β-cell developmental genes. In parallel, altered lncRNA expression profiles and extensive miRNA-lncRNA interactions highlighted the formation of ceRNA networks regulating pancreatic endocrine specification. Together, our findings unravel a novel mRNA-miRNA-lncRNA regulatory axis in governing pancreatic development and function in relation to RFX3 loss. These findings provide a valuable foundation for future studies aimed at elucidating the mechanistic contribution of ncRNAs to human pancreatic development and exploring their potentials as therapeutic targets in diabetes.

## CRediT authorship contribution statement

**Noura Aldous:** Writing – original draft, Validation, Methodology, Formal analysis, Data curation. **Aldana Alnesf:** Writing – original draft, Validation, Methodology, Formal analysis, Data curation. **Ahmed K. Elsayed:** Writing – original draft, Methodology, Formal analysis. **Bushra Yasin Abohalawa:** Writing – review & editing, Methodology, Formal analysis. **Nehad M. Alajez:** Writing – review & editing, Methodology, Formal analysis. **Essam M. Abdelalim:** Writing – review & editing, Supervision, Funding acquisition, Conceptualization.

## Data availability

The RNA-sequencing and small RNA-sequencing datasets have been submitted to the Zenodo repository and can be accessed via the following links: DOI 10.5281/zenodo.13647651 and 10.5281/zenodo.16743896, respectively.

## Funding

This work was funded by grants from 10.13039/100019475Sidra Medicine (No. SDR400217). Noura Aldous and Aldana Alnesf, co-first authors of this article, are PhD students supported by QRDI sponsorship (No. GSRA9-L-1-0511-22008 and QRLP10-G-1803010, respectively).

## Conflict of interests

The authors declared no conflict of interests.
