# Supplementary material for: RFX3 loss disrupts non-coding RNA networks in iPSC-derived pancreatic progenitors
Source: Genes Dis. 2025 Nov 27;13(4):101959. doi: 10.1016/j.gendis.2025.101959 (PMC13015203; doi:10.1016/j.gendis.2025.101959)
Supplement: Multimedia component 1 [file mmc1.pdf]

Supplementary Materials

RFX3 loss disrupts non-coding RNA networks in iPSC-derived pancreatic progenitors

Noura Aldous [a,b,1], Aldana Alnesf [a,b,1], Ahmed K. Elsayed [a,b], Bushra Yasin Abohalawa [b,c], Nehad M. Alajez [b,c], Essam M. Abdelalim [a,b*]

[a]*Pluripotent Stem Cell Disease Modelling Lab, Translational Medicine Department, Research Branch, Sidra Medicine, Doha, Qatar.*

[b]*College of Health and Life Sciences, Hamad Bin Khalifa University (HBKU), Qatar Foundation (QF), Doha, Qatar.*

[c]*Translational Oncology Research Centre (TORC), Qatar Biomedical Research Institute (QBRI), Hamad Bin Khalifa University (HBKU), Qatar Foundation (QF), Doha, Qatar.*

[1] These authors contributed equally to this work and should be considered first authors.

***Corresponding author**

Essam M. Abdelalim, PhD

E-mail: emohamed3@sidra.org

This Supplementary Material includes:

- Materials and Methods
- Figures S1-S3
- Tables S1-S4

Materials and Methods

Maintenance of iPSCs and their differentiation into pancreatic progenitors

RFX3-knockout (KO) human iPSC lines (*RFX3* KO1 and *RFX3* KO2) and their wild-type (WT) control iPSCs, were previously established and characterized in our laboratory [1]. All cell lines were maintained in mTeSR Plus medium (Stem Cell Technologies, Canada) on Matrigel-coated dishes (Corning, USA). iPSCs were differentiated *in vitro* into pancreatic progenitor cells using our established protocol [2] (Table S1).

Immunofluorescence

Cultured cells were washed once with phosphate-buffered saline (PBS), then fixed using 4% paraformaldehyde (PFA) for 20-25 minutes at room temperature. The cells were then washed three times at 10 minutes interval with tris-buffered saline containing 0.5% Tween 20 (TBST). To permeabilize the cells, PBS containing 0.5% Triton X-100 (PBST) was added for 30 minutes at room temperature. Blocking was performed overnight at 4°C in PBST containing 6% bovine serum albumin (BSA). Primary antibody incubation was carried out overnight at 4°C in PBST containing 3% BSA, followed by three washes with TBST. The cells were then incubated for 1 hour at room temperature with PBST-diluted secondary antibodies at a dilution of 1:500. Nuclei were then stained using Hoechst 33258 (1:5000 in PBS) for 5 minutes, followed by three washes with PBS. Imaging was performed using Cell Discoverer 7. Primary antibody list is provided in Table S2.

Western blotting

A single well of a 6-well plate was used for protein extraction using RIPA lysis buffer supplemented with protease inhibitors. For protein quantification, Pierce BCA Protein Assay Kit (ThermoFisher Scientific, USA) was used. 20 µg of total protein was loaded on SDS-PAGE gels and transferred onto PVDF membranes. Membranes were then blocked with 15% skimmed milk in TBST incubated at room temperature for 2-3 hours. Primary antibodies diluted in 5% skimmed milk in TBST were added overnight at 4°C then washed using TBST. Horseradish peroxidase-conjugated secondary antibodies (Jackson ImmunoResearch) were applied at a dilution of 1:10,000 for 1 hour at room temperature, followed by additional TBST washes. Membranes were developed using the SuperSignal West Pico Chemiluminescent Substrate (ThermoFisher Scientific) and imaged with the iBright™ CL 1000 Imaging System (Invitrogen). Details of primary antibodies are provided in Table S2.

RNA extraction and RT-qPCR expression analysis

Total RNA was isolated from a single well of a 6-well plate using the Direct-zol™ RNA MiniPrep Kit (Zymo Research, USA). For lncRNA expression analysis, complementary DNA (cDNA) was synthesized from 1 µg of RNA using High-Capacity cDNA Reverse Transcription Kit (Applied Biosystems) following manufacturer's protocol. Real-time quantitative PCR (RT-qPCR) was performed in replicates using GoTaq qPCR SYBR Green Master Mix (Promega, USA). The fold change $2^{-\Delta\Delta Ct}$ method was used to calculate the relative lncRNA expression, with GAPDH serving as the endogenous control. LncRNA primer sequences are provided in Table S3.

5 ng/ul of total RNA was used for miRNA reverse transcription reaction using miRCURY LNA RT Kit (QIAGEN, Cat. 339340). Relative miRNA expression level quantification was done using specific miRCURY LNA miRNA PCR Assays and miRCURY LNA SYBR® Green PCR Kit (QIAGEN, Cat. 339345) while diluting the reverse transcription reaction to 1:20. Relative miRNA expression was calculated using the fold change $2^{-\Delta\Delta Ct}$ method, with SNORD48 as the endogenous control for normalization. Used miRCURY LNA miRNA PCR Assays are listed in Table S3.

Bulk RNA sequencing and differential lncRNA expression analysis

RNA sequencing (RNA-seq) data were processed as described previously [3]. A total 1 µg of RNA was used to isolate mRNA with the NEBNext Poly(A) mRNA Magnetic Isolation Kit (E7490, New England Biolabs, Massachusetts, USA). RNA samples with RIN values ≥ 7.0 were selected for library preparation using the NEBNext Ultra Directional RNA Library Prep Kit (E7420L, New England Biolabs, Massachusetts, USA). All libraries passed quality check and were sequenced on an Illumina NextSeq 2000 platform, yielding an average of 50 million reads per sample. FASTQ files were generated using Illumina BCL2Fastq Conversion Software v2.20. Paired-end FASTQ files were aligned to the GRCh38 reference genome (> 99% mapping) using the built-in module and default settings of CLC Genomics Workbench v24.0.2. Transcript expression levels, quantified as Transcripts Per Million (TPM), were imported into AltAnalyze v2.1.3 [4] for differential expression analysis, following our previously described pipeline [5]. A cut-off criterion of $-0.5 > \log_2$ [fold change] > 0.5 and p-value < 0.05 was used for differentially expressed lncRNAs (DElncRNAs).

miRNA sequencing and differential miRNA expression analysis

A total of 1 µg of RNA was used for miRNA library preparation using NEBNext Multiplex Small RNA library Prep Set for Illumina while following manufacturer's protocol (E7560S, New England BioLabs, USA). Resulting amplified cDNA constructs were then purified using Monarch PCR

purification kit (T1130S, New England BioLabs, USA). Libraries that passed quality check were then sequenced on an Illumina NextSeq 2000 platform, yielding an average of 20 million reads per sample. For miRNA expression analysis, FASTQ files were aligned to the miRBase v22 database (mature miRNAs), and miRNA expression was quantified in terms of total read counts using the small RNA analysis workflow with default settings in CLC Genomics Workbench 20.2, as described previously [6]. Most of the annotated reads were successfully mapped to the miRBase v22 database. Only mature miRNAs (5p and 3p) were considered for analysis in accordance with miRBase v22. To ensure accurate miRNA quantification, count reads were normalized using the TMM (trimmed mean of M values) method, and the resulting Log_2-transformed Counts per Million (CPM) values were used for subsequent differential expression analysis. Differentially expressed miRNAs (DEmiRs) in *RFX3* KO PPs, compared to WT-PPs, were identified based on $-0.5 > \log_2$ [fold change] > 0.5 and *p*-value threshold of < 0.05.

DEmiRs target prediction analysis and competing endogenous RNA network generation

For miRNA-mRNA target prediction analysis, DEmiR and DEG lists were uploaded to Ingenuity Pathway Analysis (IPA) software (QIAGEN, Germany). MiRNA target filter with default settings were used with a significance threshold of $p < 0.05$ and $\log_2$ [fold change] < -0.5 or > 0.5. For miRNA-lncRNA target prediction analysis, miRNA targets were identified by investigating the sequence complementarity between each miRNA sequence and the corresponding lncRNA transcript sequences (i.e. upregulated miRNAs vs downregulated lncRNAs and vice versa) from the Human Gencode (R) 47 [7] using the miRanda algorithm (v3.3a) [8]. Briefly, the algorithm was used to align known miRNA sequences against the corresponding lncRNA transcripts using multiple alignment score thresholds; both Watson-Crick (|) or GU wobble base pairing with stringent alignment scores (≥140) and thermodynamic parameters with a high binding energy threshold (≤–1 kcal/mol) were applied to identify the miRNA targets with high binding to lncRNA sequences. For ceRNA network, identified miRNA target lists were used for miRNA-mRNA-lncRNA network generation using Cytoscape 3.10.3 software (National Resource for Network Biology).

Statistical analysis

At least three biological replicates were analysed for each experiment, and *p*-values were calculated using an ANOVA test on GraphPad Prism 8 software (GraphPad Software, Boston, MA, USA; www.graphpad.com). Data are represented as mean ± SD.

Supplementary Figures

Figure S1

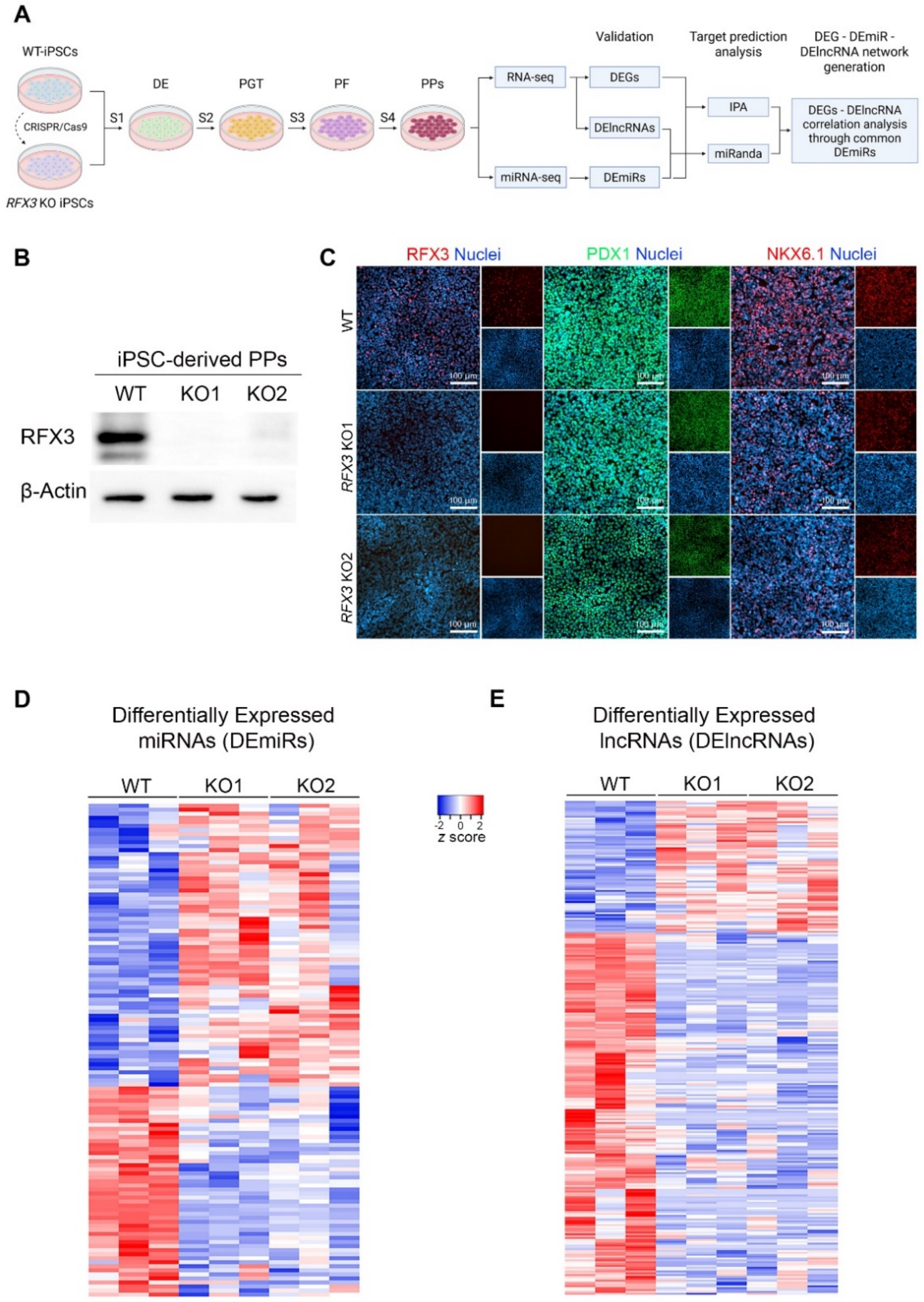

Figure S1. Effect of RFX3 loss on pancreatic progenitors (PPs). (A) Schematic representation of the experimental workflow, including iPSC differentiation into pancreatic progenitors (PPs). (B) Western blot analysis confirming the absence of RFX3 protein in PPs derived from *RFX3* KO iPSCs compared to WT-iPSCs. (C) Immunofluorescence showing loss of RFX3 expression and the effect of RFX3 deficiency on key PP markers, PDX1 and NKX6.1. Heatmaps showing the global expression profile of differentially expressed miRNAs (DEmiRs) (D) and lncRNAs (DElncRNAs) (E) in *RFX3* KO PPs (*n*=3). DE; definitive endoderm, PGT; primitive gut tube, PF; posterior foregut, PPs; pancreatic progenitors.

Figure S2

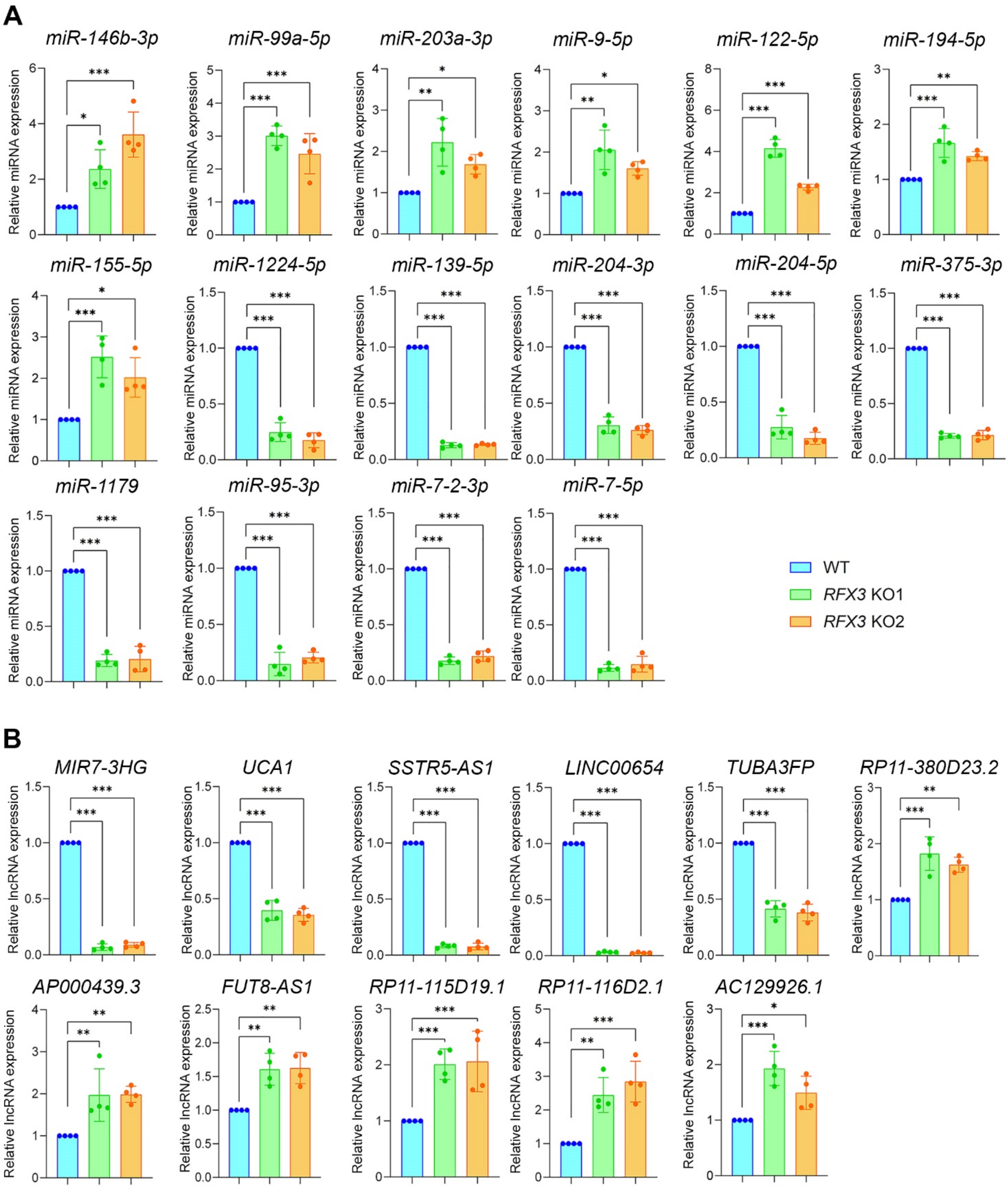

Figure S2. Non-coding RNA-seq validation of selected dysregulated miRNAs and lncRNAs. RT-qPCR validation of selected upregulated and downregulated differentially expressed miRNAs (DEmiRs) (A) and differentially expressed lncRNAs (DElncRNAs) (B) (*n*=4). Data are represented as mean ± SD. **p*<0.05, ***p*<0.01, ****p*<0.001.

Figure S3

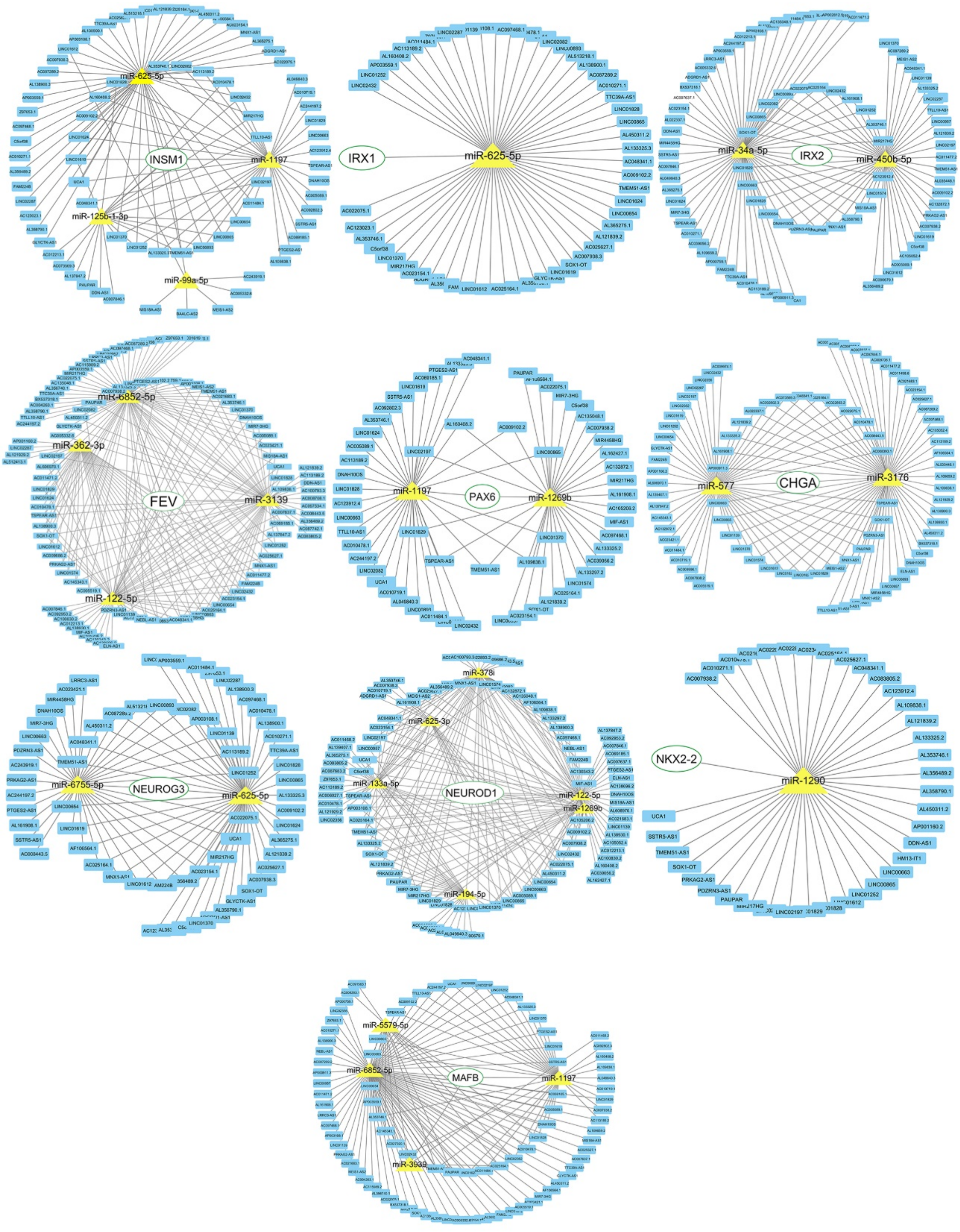

Figure S3. Representative miRNA-mRNA-lncRNA correlation networks in RFX3-deficient pancreatic progenitors. The networks illustrate predicted interactions between DEmiRs, DElncRNAs, and DEGs. miRNA target prediction was performed using Ingenuity Pathway Analysis (IPA) and miRanda for DEGs and DElncRNAs, respectively. Selected pancreatic progenitor markers were used to construct ceRNA networks. Networks were generated using Cytoscape software.

Supplementary Tables

Table S1. Media formulation and details of cytokine added for *in vitro* differentiation protocol.

Differentiation stage	Media supplement	Cytokines
Stage 1 (DE)<br>3 days	**MCDB 131**:<br>1% Pen/Strep<br>1% L-Glutamine<br>0.5% Fatty acid free BSA<br>1.5 g/L $NaHCO_3$<br>10 mM D-Glucose	**Day 1**:<br>1 µM Y-27632 (day 1 only)<br>2 µM CHIR99021 (day 1 only)<br>100 mM Activin A<br>0.25 mM Vitamin C
Stage 2 (PGT)<br>2 days	**MCDB 131**:<br>1% Pen/Strep<br>1% L-Glutamine<br>0.5% Fatty acid free BSA<br>1.5 g/L $NaHCO_3$<br>10 mM D-Glucose	50 ng/mL FGF-10<br>0.75 µM Dorsomorphin<br>3 ng/mL WNT-3a<br>0.25 mM Vitamin C
Stage 3 (PF)<br>2 days	**DMEM**:<br>1% Pen/Strep<br>4.5 g/L Glucose<br>110 mg/L Sodium pyruvate	50 ng/mL FGF-10<br>200 nM LDN193189<br>0.25 µM SANT-1<br>2 µM Retinoic acid<br>0.25 mM Vitamin C<br>1% B27 w/o Vitamin A
Stage 4 (PPs)<br>4 days	**DMEM**:<br>1% Pen/Strep<br>4.5 g/L Glucose<br>110 mg/L Sodium pyruvate	100 ng/mL EGF<br>10 mM Nicotinamide<br>200 nM LDN193189<br>0.25 mM Vitamin C<br>1% B27 w/o Vitamin A

Table S2. List of used antibodies.

Antibody	Catalog number	Company	RRID	Dilution
Anti-β-Actin	Sc-47778	Santa Cruz Biotechnology	AB_626632	1:10,000 (WB)
Anti-PDX1	ab47308	Abcam	AB_777178	1:1000 (IF)
Anti-NKX6.1	F55A12	DSHB	AB_532379	1:2000 (IF)
Anti-RFX3	NBP1-86301	Novus Biologicals	AB_11019457	1:1000 (IF),<br>1:500 (WB)
Alexa Fluor 568 anti-rabbit IgG	A10042	Invitrogen	AB_2534017	1:500 (IF)
Alexa Fluor 594 anti-mouse IgG	A32744	Invitrogen	AB_2762826	1:500 (IF)
Alexa Fluor 488 anti-guinea pig IgG	A11073	Invitrogen	AB_2534117	1:500 (IF)
Peroxidase AffiniPure Donkey anti-Rabbit IgG (H+L)	711-035-152	Jackson ImmunoResearch Laboratories	AB_10015282	1:10,000 (WB)
Peroxidase AffiniPure Donkey anti-Mouse IgG (H+L)	715-035-150	Jackson ImmunoResearch Laboratories	AB_2340770	1:10,000 (WB)

Table S3. List of used RT-qPCR primers and assays.

**LncRNA primers list**		
**LncRNA**	**Forward Primer**	**Reverse Primer**
MIR7-3HG	TGGACATTTTCCCAGAAAGG	CAGGGTTCTGGAGACGAAAC
UCA1	CTTCTGCATAGGATCTGCAATCAG	TTTTGTCCCCATTTTCCATCATACG
SSTR5-AS1	ACTACAGGTGCCATCAGACC	AGCCTGCCATCCTAACACTT
LINC00654	CTGGTCAGGTGCTCTACCAA	AGCAAAACCCCACACCAATAAC
TUBA3FP	CCTGCTGCATGTTGTACAGA	TAAATCCAATCGGACACCAGT
RP11-380D23.2	ACCATACCCCTTCCTCCAGTCCCT	TCTATCCGTCTGTCCTGGGAGAGA
RP11-116D2.1	ATGGGTGGGTGAGCGAATAA	TCCAGGCCTCCTTTCAGTTT
RP11-115D19.1	TAAAACCTGCAAATTCACATCTTC	AAGTAGGTAAGTAGGGCAGTGCAT
AP000439.3	CCCCAGGCTAGGAAGATGT	GAGCCAAGAGGTCCTCACAG
FUT8-AS1	GGCTCCTTGCTACTTTTAGGG	TGGGGGGGGTCTTTCTCTTC
AC129926.1	GAATGATGGTGCCAGTCAAGTC	TCTGTAGTGTGTTTCAGCCACTG
GAPDH	ACGACCACTTTGTCAAGCTCATTTC	GCAGTGAGGGTCTCTCTCTTCCTCT

**miRNA Assays**	
**miRNA PCR Assay**	**Assay ID**
hsa-miR-95-3p	YP00204288
hsa-miR-7-2-3p	YP00204581
hsa-miR-7-5p	YP02119317
hsa-miR-1224-5p	YP02108797
hsa-miR-139-5p	YP00205874
hsa-miR-204-5p	YP00206072
hsa-miR-204-3p	YP02113689
hsa-miR-375-3p	YP00204362
hsa-miR-1179	YP00204624
hsa-miR-146b-3p	YP02119309
hsa-miR-99a-5p	YP00204521
hsa-miR-203a-3p	YP00205914
hsa-miR-9-5p	YP00204513
hsa-miR-122-5p	YP00205664
hsa-miR-194-5p	YP00204080
hsa-miR-155-5p	YP02119311

Table S4. Selected upregulated differentially expressed miRNAs predicted to target key downregulated pancreatic-related genes in *RFX3* KO PPs compared to WT-PPs ($\log_2$ [fold change] > 0.5, $p < 0.05$).

Upregulated miRNA	$\log_2$ [fold change]	*p*-value	Target gene	$\log_2$ [fold change]	*Adj p*-value
miR-584-5p	0.800	6.66×10^{-3}	*GCG*	-9.432	0
miR-181a-2-3p	1.081	2.19×10^{-3}	*UCN3*	-8.572	1.39×10^{-14}
miR-3139	0.704	2.04×10^{-2}			
miR-3085-3p	0.852	3.69×10^{-4}	*SST*	-6.520	7.25×10^{-184}
miR-625-5p	0.614	2.20×10^{-2}			
miR-7974	1.260	1.79×10^{-2}	*KCNK16*	-5.536	6.51×10^{-58}
miR-1269b	0.874	1.30×10^{-3}			
miR-6852-5p	0.536	3.84×10^{-2}			
miR-122-5p	1.108	1.83×10^{-3}	*ARX*	-5.187	4.51×10^{-58}
miR-125b-1-3p	0.987	1.08×10^{-2}			
miR-34a-5p	0.816	4.55×10^{-3}			
miR-9-5p	0.774	3.36×10^{-2}			
miR-4755-5p	0.561	1.89×10^{-2}			
miR-34a-5p	0.816	4.55×10^{-3}	*ISL1*	-4.288	9.78×10^{-66}
miR-625-5p	0.614	2.20×10^{-2}			
miR-450b-5p	0.592	4.03×10^{-2}			
miR-1197	0.548	2.66×10^{-2}			
miR-625-5p	0.614	2.20×10^{-2}	*IRX1*	-3.886	1.31×10^{-11}
miR-3939	0.682	3.83×10^{-2}	*MAFB*	-3.823	1.42×10^{-61}
miR-5579-5p	0.632	1.38×10^{-2}			
miR-1197	0.548	2.66×10^{-2}			
miR-6852-5p	0.536	3.84×10^{-2}			
miR-181a-2-3p	1.081	2.19×10^{-3}	*KCNH6*	-3.651	3.39×10^{-41}
miR-1269b	0.874	1.30×10^{-3}	*PAX6*	-3.532	4.98×10^{-14}
miR-1197	0.548	2.66×10^{-2}			
miR-3085-3p	0.852	3.69×10^{-4}	*PTPRN*	-3.492	4.23×10^{-48}
miR-1269b	0.874	1.30×10^{-3}	*SSTR2*	-3.228	1.60×10^{-51}
miR-125b-5p	0.864	2.72×10^{-2}			
miR-34a-5p	0.816	4.55×10^{-3}			
miR-99a-5p	1.271	2.17×10^{-4}	*PTPRN2*	-3.194	1.19×10^{-82}
miR-3085-5p	0.980	5.58×10^{-3}	*GCK*	-3.051	4.59×10^{-15}
miR-1269b	0.874	1.30×10^{-3}			
miR-3085-3p	0.852	3.69×10^{-4}			
miR-4515	0.800	4.23×10^{-2}			
miR-3085-3p	0.852	3.69×10^{-4}	*PCSK1N*	-2.892	1.08×10^{-20}
miR-34a-5p	0.816	4.55×10^{-3}	*ASCL1*	-2.890	7.40×10^{-9}
miR-625-5p	0.614	2.20×10^{-2}			
miR-625-5p	0.614	2.20×10^{-2}	*KCNK17*	-2.808	1.14×10^{-10}
miR-215-5p	1.698	4.48×10^{-3}	*FFAR2*	-2.777	1.95×10^{-13}
miR-34a-5p	0.816	4.55×10^{-3}			

miR-3176	0.718	6.29×10^{-3}			
miR-3139	0.704	2.04×10^{-2}			
miR-301b-5p	0.595	7.55×10^{-4}			
miR-221-5p	0.542	2.15×10^{-3}			
miR-18a-5p	0.541	2.33×10^{-2}			
miR-215-5p	1.698	4.48×10^{-3}	*ERO1B*	-2.535	5.07×10^{-42}
miR-122-3p	1.391	1.35×10^{-3}			
miR-6852-5p	0.536	3.84×10^{-2}			
miR-122-5p	1.108	1.83×10^{-3}	*NEUROD1*	-2.455	2.66×10^{-31}
miR-1269b	0.874	1.30×10^{-3}			
miR-378i	0.657	9.41×10^{-3}			
miR-133a-5p	0.555	2.02×10^{-2}			
miR-194-5p	0.548	4.82×10^{-2}			
miR-625-3p	0.522	9.85×10^{-3}			
miR-181a-3p	1.081	3.54×10^{-2}	*POU2F2*	-2.235	1.01×10^{-9}
miR-3085-5p	0.98	5.58×10^{-3}			
miR-3085-3p	0.852	3.69×10^{-4}			
miR-9-5p	0.774	3.36×10^{-2}			
miR-625-5p	0.614	2.20×10^{-2}			
miR-4755-5p	0.561	1.89×10^{-2}			
miR-221-5p	0.542	2.15×10^{-3}			
miR-6852-5p	0.536	3.84×10^{-2}			
miR-3176	0.718	6.29×10^{-3}	*CHGA*	-2.230	9.70×10^{-17}
miR-577	0.504	1.58×10^{-2}			
miR-34a-5p	0.816	4.55×10^{-3}	*IRX2*	-2.197	1.41×10^{-12}
miR-450b-5p	0.592	4.03×10^{-2}			
miR-122-5p	1.108	1.83×10^{-3}	*FEV*	-2.111	1.68×10^{-16}
miR-362-3p	0.772	2.24×10^{-4}			
miR-3139	0.704	2.04×10^{-2}			
miR-6852-5p	0.536	3.84×10^{-2}			
miR-6755-5p	0.917	1.58×10^{-3}	*NEUROG3*	-1.972	1.29×10^{-18}
miR-625-5p	0.614	2.20×10^{-2}			
miR-1290	0.605	2.55×10^{-2}	*NKX2.2*	-1.960	9.99×10^{-34}
miR-99a-5p	1.271	2.17×10^{-4}	*INSM1*	-1.939	1.01×10^{-18}
miR-125b-1-3p	0.987	1.08×10^{-2}			
miR-625-5p	0.614	2.20×10^{-2}			
miR-1197	0.548	2.66×10^{-2}			
miR-125b-1-3p	0.987	1.08×10^{-2}	*SSTR1*	-1.922	2.57×10^{-18}
miR-3939	0.682	3.83×10^{-2}			
miR-301b-5p	0.595	7.55×10^{-4}			
miR-99a-5p	1.271	2.17×10^{-4}	*LMX1B*	-1.873	1.76×10^{-56}
miR-7974	1.26	1.79×10^{-2}			
miR-122-5p	1.108	1.83×10^{-3}			

miR-3085-5p	0.98	5.58×10^{-3}			
miR-3085-3p	0.852	3.69×10^{-4}			
miR-3176	0.718	6.29×10^{-3}			
miR-625-5p	0.614	2.20×10^{-2}			
miR-221-5p	0.542	2.15×10^{-3}			
miR-18a-5p	0.541	2.33×10^{-2}			
miR-6852-5p	0.536	3.84×10^{-2}			
miR-125b-1-3p	0.987	1.08×10^{-2}	*PCSK2*	-1.613	5.29×10^{-4}
miR-4515	0.800	4.23×10^{-2}			
miR-9-5p	0.774	3.36×10^{-2}			
miR-625-5p	0.614	2.20×10^{-2}			
miR-362-3p	0.772	2.24×10^{-4}	*SCGN*	-1.565	1.89×10^{-25}
miR-1270	0.609	2.06×10^{-2}			
miR-125b-1-3p	0.987	1.08×10^{-2}	*SSTR3*	-1.499	9.73×10^{-4}
miR-125b-5p	0.864	2.72×10^{-2}			
miR-3176	0.718	6.29×10^{-3}			
miR-1197	0.548	2.66×10^{-2}			
miR-221-5p	0.542	2.15×10^{-3}			
miR-6852-5p	0.536	3.84×10^{-2}			
miR-122-5p	1.108	1.83×10^{-3}	*GP2*	-1.424	2.81×10^{-7}
miR-125b-5p	0.864	2.72×10^{-2}			
miR-215-3p	1.234	2.58×10^{-2}	*ZNF506*	-1.420	2.77×10^{-15}
miR-3085-3p	0.852	3.69×10^{-4}	*STX1A*	-1.327	4.86×10^{-23}
miR-34a-5p	0.816	4.55×10^{-3}			
miR-3176	0.718	6.29×10^{-3}			
miR-625-5p	0.614	2.20×10^{-2}			
miR-6852-5p	0.536	3.84×10^{-2}			
miR-6755-5p	0.917	1.58×10^{-3}	*CPA4*	-1.312	6.79×10^{-6}
miR-125b-5p	0.864	2.72×10^{-2}			
miR-9-5p	0.774	3.36×10^{-2}			
miR-362-3p	0.772	2.24×10^{-4}			
miR-5579-5p	0.632	1.38×10^{-2}			
miR-412-3p	0.58	1.73×10^{-2}			
miR-1197	0.548	2.66×10^{-2}			
miR-3176	0.718	6.29×10^{-3}	*PTF1A*	-1.156	2.82×10^{-3}
miR-18a-5p	0.541	2.33×10^{-2}			
miR-363-3p	0.530	1.91×10^{-3}			
miR-577	0.504	1.58×10^{-2}			
miR-34a-5p	0.816	4.55×10^{-3}	*KCNJ11*	-1.152	5.83×10^{-4}
miR-4473	0.698	3.46×10^{-2}			
miR-221-5p	0.542	2.15×10^{-3}			
miR-215-5p	1.698	4.48×10^{-3}	*RFX6*	-1.144	1.63×10^{-11}
miR-181a-5p	0.855	1.67×10^{-2}			

miR-194-5p	0.548	4.82×10^{-2}			
miR-122-3p	1.391	1.35×10^{-3}	*WNT9A*	-1.089	2.96×10^{-4}
miR-181a-5p	0.855	1.67×10^{-2}			
miR-4515	0.800	4.23×10^{-2}			
miR-362-3p	0.772	2.24×10^{-4}			
miR-3176	0.718	6.29×10^{-3}			
miR-378i	0.657	9.41×10^{-3}			
miR-6852-5p	0.536	3.84×10^{-2}			

References

1. Memon B, Aldous N, Elsayed AK, Ijaz S, Hayat S, Abdelalim EM. RFX3 is essential for the generation of functional human pancreatic islets from stem cells. *Diabetologia.* 2025.
2. Memon B, Karam M, Al-Khawaga S, Abdelalim EM. Enhanced differentiation of human pluripotent stem cells into pancreatic progenitors co-expressing PDX1 and NKX6.1. *Stem Cell Research & Therapy.* 2018;9(1):15.
3. Aldous N, Elsayed AK, Memon B, Ijaz S, Hayat S, Abdelalim EM. Deletion of RFX6 impairs iPSC-derived islet organoid development and survival, with no impact on PDX1(+)/NKX6.1(+) progenitors. *Diabetologia.* 2024.
4. Emig D, Salomonis N, Baumbach J, Lengauer T, Conklin BR, Albrecht M. AltAnalyze and DomainGraph: analyzing and visualizing exon expression data. *Nucleic Acids Res.* 2010;38(Web Server issue):W755-762.
5. Shaath H, Vishnubalaji R, Elango R, Velayutham D, Jithesh PV, Alajez NM. Therapeutic targeting of the TPX2/TTK network in colorectal cancer. *Cell Commun Signal.* 2023;21(1):265.
6. Elango R, Rashid S, Vishnubalaji R, et al. Transcriptome profiling and network enrichment analyses identify subtype-specific therapeutic gene targets for breast cancer and their microRNA regulatory networks. *Cell Death Dis.* 2023;14(7):415.
7. Frankish A, Diekhans M, Ferreira AM, et al. GENCODE reference annotation for the human and mouse genomes. *Nucleic Acids Res.* 2019;47(D1):D766-d773.
8. John B, Enright AJ, Aravin A, Tuschl T, Sander C, Marks DS. Human MicroRNA Targets. *PLOS Biology.* 2004;2(11):e363.
